# Upregulation of SOCS-3 and PIAS-3 Impairs IL-12-Mediated Interferon-Gamma Response in CD56^+^ T Cells in HCV-Infected Heroin Users

**DOI:** 10.1371/journal.pone.0009602

**Published:** 2010-03-09

**Authors:** Li Ye, Xu Wang, David S. Metzger, Eric Riedel, Luis J. Montaner, Wenzhe Ho

**Affiliations:** 1 Department of Pathology and Laboratory Medicine, Temple University School of Medicine, Philadelphia, Pennsylvania, United States of America; 2 Department of Psychiatry, The Center for Studies of Addiction, University of Pennsylvania School of Medicine, Philadelphia, Pennsylvania, United States of America; 3 Department of Pediatrics, Joseph Stokes, Jr. Research Institute, The Children's Hospital of Philadelphia, Philadelphia, Pennsylvania, United States of America; 4 The Wistar Institute, Philadelphia, Pennsylvania, United States of America; University of Nebraska, United States of America

## Abstract

**Background:**

CD56^+^ T cells are abundant in liver and play an important role in host innate immunity against viral infections, including hepatitis C virus (HCV) infection, a common infection among heroin abusers. We thus investigated the *in vivo* impact of heroin use or heroin use plus HCV infection on the CD56^+^ T cell frequency and function.

**Methodology/Principal Findings:**

A total of 37 heroin users with (17) or without (20) HCV infection and 17 healthy subjects were included in the study. Although there was no significant difference in CD56^+^ T cell frequency in PBMCs among three study groups, CD56^+^ T cells isolated from the heroin users had significantly lower levels of constitutive interferon-gamma (IFN-γ) expression than those from the normal subjects. In addition, when stimulated by interleukin (IL)-12, CD56^+^ natural T cells from HCV-infected heroin users produced significantly lower levels of IFN-γ than those from the normal subjects. This diminished ability to produce IFN-γ by CD56^+^ T cells was associated with the increased plasma HCV viral loads in the HCV-infected heroin users. Investigation of the mechanisms showed that although heroin use or heroin use plus HCV infection had little impact on the expression of the key positive regulators (IL-12 receptors, STAT-1, 3, 4, 5, JAK-2, and TYK-2) in IL-12 pathway, heroin use or heroin use plus HCV infection induced the expression of suppressor of cytokine signaling protein-3 (SOCS-3) and protein inhibitors of activated STAT-3 (PIAS-3), two key inhibitors of IL-12 pathway.

**Conclusion/Significance:**

These findings provide compelling *in vivo* evidence that heroin use or heroin use plus HCV infection impairs CD56^+^ T cell-mediated innate immune function, which may account for HCV infection and persistence in liver.

## Introduction

Hepatitis C virus (HCV) has now been recognized as a major public health problem worldwide. HCV infection is a significant cause of chronic liver disease, with frequent progression to cirrhosis and an elevated risk for the development of hepatocellular carcinoma. In the United States, about 15–30% of all HIV-infected persons are also infected with HCV. Since the use of highly active antiretroviral therapy in 1996, HCV-related liver disease has now emerged as a major cause of morbidity and mortality among HIV-infected patients. HCV infection is extremely common among injection heroin users [1], [2], [3], [4], [5], [6]. Rates of HCV infection among past and current injection drug users are extremely high generally ranging from 70 to over 90% (antibody positive for HCV) in the United States [7], [8], [9], [10]. Drug abuse, especially the abuse of heroin, the most commonly used opiate, is a significant risk factor for HCV infection and the development of chronic HCV disease [1], [2], [3], [4], [5], [6].

The negative impact of drug abuse on host immune system has been currently considered as an important factor in increasing the likelihood for HCV infection and the development of chronic HCV disease in drug abuse population. Opioid drugs, such as heroin and morphine, have been demonstrated to impair the immune system [11], [12], [13] and facilitate HCV replication in human hepatocytes [14], [15]. Opioids alter immune system by acting directly on immune cells, possibly via opioid receptor on the surface of immune cells [16]. Opioids exert profound influence on function of the immune cells, including T cells, B cells, monocytes, and NK cells. Opioids have been shown to inhibit the expression of antiviral cytokines, including interferon (IFN)- α/β and IFN-γ in PBMCs [17], [18], in T cells [19] and monocytes [20]. However, it is still unclear whether opioids such as heroin suppress CD56^+^ T cell-mediated innate immunity against HCV infection. Since CD56^+^ T cells are abundant in liver and are a key member of host innate immune cell family in protecting liver from viral infections, the impairment of CD56^+^ T cell-mediated innate immunity may account for HCV infection and persistence in liver.

CD56^+^ T cells express both natural killer (NK) and T cell markers (CD56 and CD3, respectively) and functionally display properties of both NK cells and T cells [21], [22]. A normal human liver, as the primary site of HCV infection, contains lymphocytes that are enriched for CD56^+^ T cells [23], [24]. CD56^+^ T cells possess the ability to rapidly produce large quantities of both Th1 and Th2 cytokines, particularly IFN-γ, tumor necrosis factor-α, interleukin(IL) -2, IL-4, and IL-13 without need for priming or clonal expansion [22], [23], [25], [26], [27], [28]. This ability of CD56^+^ T cells permits them to play a key role in the communications between the innate and adaptive immune cells. CD56^+^ T cells alongside NK cells as well as NKT cells have been considered as frontline innate immune effectors and potential regulators for both innate and adaptive immune responses against microorganisms including viruses [24], [29], [30]. More importantly, recent studies have highlighted that CD56^+^ T cells play an important role in determining the outcome of acute HCV infection [31], and are known to be depleted in the livers of patients with chronic HCV infection [31], [32], [33], [34]. Our recent study [35] also showed that CD56^+^ T cells have the ability to inhibit HCV replication in human hepatocytes *in vitro*.

Since the liver contains a large number of CD56^+^ T cells [23], [24], an important question is why CD56^+^ T cells in liver fail to protect hepatocytes from HCV infection and eliminate the virus in the liver. One speculation is that heroin use or heroin use plus HCV infection impair CD56^+^ T cell-mediated antiviral function. It is known that HCV has ability to escape and counteract host innate immunity. In addition, drugs of use, such as heroin, have been shown to suppress the host immune system. In the present study, we investigated the frequency and antiviral function of circulating CD56^+^ T cells isolated from the heroin users with or without HCV infection. We also examined the mechanisms underlying the impact of heroin use or heroin use plus HCV infection on the antiviral function of CD56^+^ T cells.

## Results

### 1. Effect of Heroin Use/HCV Infection on CD56^+^ T Cell Frequency in PBMCs

**Figure 1:**
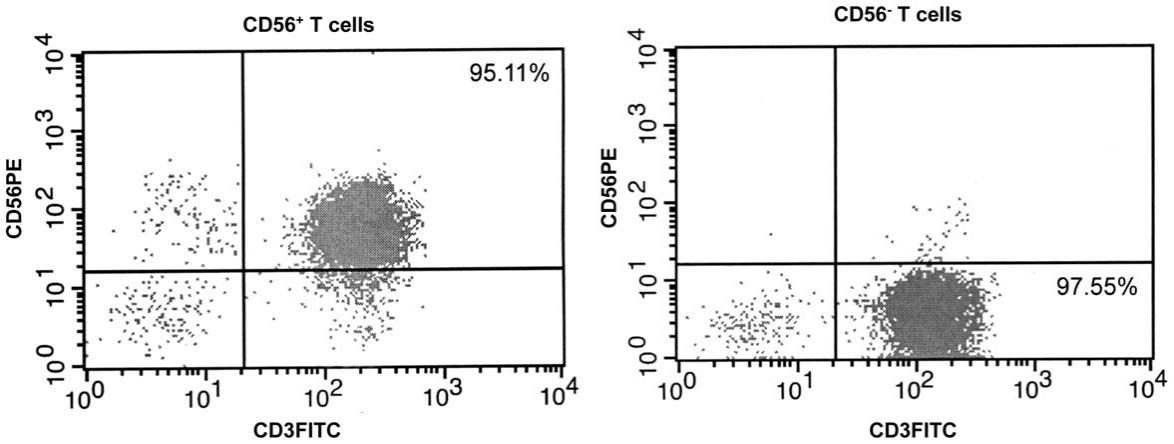
Isolation of CD56^+^ T and CD56^-^ T cells from PBMCs. The purity of enriched cell population was determined by flow cytometry using antibodies to CD56 (PE) and CD3 (FITC) markers.

**Figure 2:**
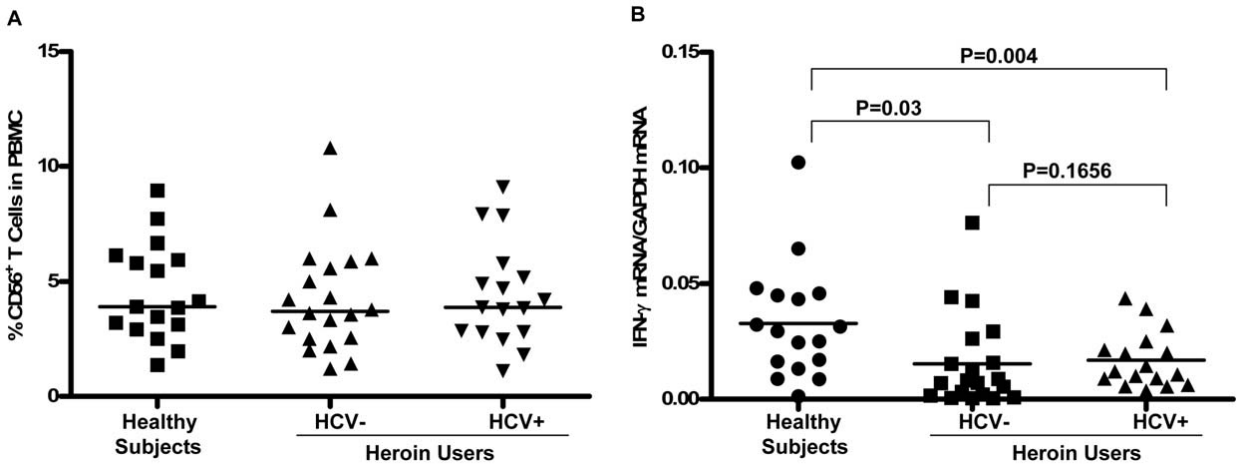
Effect of heroin use or heroin use plus HCV infection on CD56^+^ T cells. (A) Heroin use or heroin use plus HCV infection has little impact on CD56^+^ T cell frequency (%) in PBMCs. The CD56^+^ T cell frequency (%) in PBMCs from healthy subjects (n = 17), heroin users with (n = 17) or without (n = 20) HCV infection were shown by scatter plots. Median values are indicated by horizontal bars. Statistical significance was calculated by the Mann-Whitney *U*-test. There is no significant difference in CD56^+^ T cell numbers in PBMCs between normal subjects, heroin users with or without HCV infection (P>0.05). (B) Heroin use down-regulates IFN-γ gene expression in unstimulated CD56^+^ T cells. CD56^+^ T cells were isolated from healthy subjects (n = 17) and heroin users with (n = 17) or without (n = 20) HCV infection. Total cellular RNA extracted from freshly isolated CD56^+^ T cells was subjected to the real-time RT PCR for IFN-γ and GAPDH mRNA quantification. The data are expressed as the relative ratio of the IFN-γ mRNA levels to GAPDH mRNA levels. Median values are indicated by horizontal bars and statistical significance was calculated by the Mann-Whitney *U*-test.

We first examined the frequency of CD56^+^ T cells in peripheral blood mononuclear cells (PBMCs) of the heroin users with or without HCV infection compared with that of the healthy subjects. CD56^+^ T cells were isolated from PBMCs by CD3^+^CD56^+^ T Cell Isolation Kit (Miltenyi Biotec, Auburn, CA) (Fig. 1). The percentages of CD56^+^ T cells in PBMCs from three study groups are shown in Fig. 2A. The frequency of circulating CD56^+^ T cells was 3.87% (median, ranging from 1.37%–8.94%) in PBMCs from the healthy subjects. Similar frequency was observed in the heroin users with (median 3.78%, ranging from 1.2%–10.8%) or without (median 3.8%, ranging from 1.11%–9.09%) HCV infection. Statistically, there were no significant differences in the percentage of CD56^+^ T cells in PBMCs among the three study groups (P>0.05).

### 2. Effect of Heroin Use or Heroin Use plus HCV Infection on IFN-γ Expression by CD56^+^ T Cells

**Figure 3:**
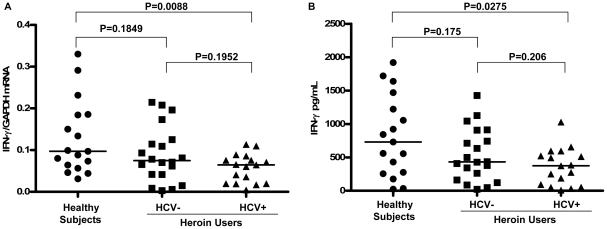
Heroin use or heroin use plus HCV infection down-regulate IFN-γ expression in IL-12-stimulated CD56^+^ T cells. CD56^+^ T cells isolated from the healthy subjects (n = 17) and the heroin users with (n = 17) or without (n = 20) HCV infection were stimulated by IL-12 *in vitro* for 48 h. (A) Total cellular RNA extracted from IL-12-stimulated CD56^+^ T cells was subjected to the real-time RT PCR for the mRNA levels of IFN-γ and GAPDH. The data are expressed as the relative ratio of the IFN-γ to GAPDH mRNA levels. Mean values are indicated by horizontal bars and statistical significance was calculated by the Mann-Whitney *U*-test. (B) IFN-γ protein levels in IL-12-stimulated CD56^+^ T cell cultures were determined by ELISA. Median values are indicated by horizontal bars and statistical significance was calculated by the Mann-Whitney *U*-test.

**Figure 4:**
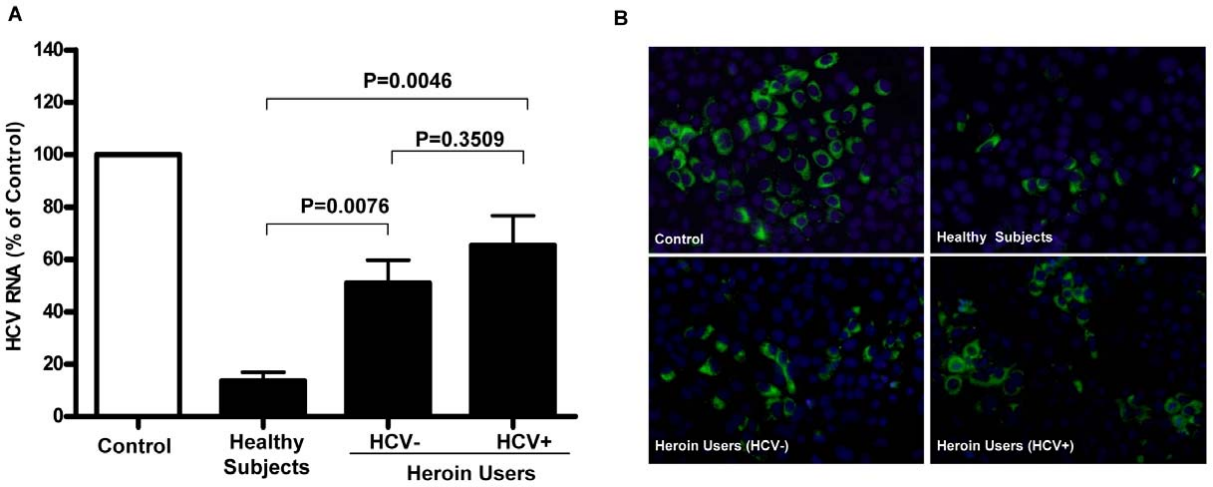
Comparison of anti-HCV activity of CD56^+^ T cells among three study groups. HCV JFH-1-infected Huh 7 cells (day 3 post infection) were cultured for 48h in the presence or absence of pooled SN from IL-12-stimulated CD56^+^ T cells from the healthy subjects and the heroin users with or without HCV infection as indicated. (A) Real-time RT-PCR analysis of HCV RNA. Total cellular RNA extracted from Huh7 cells treated with or without pooled SN of CD56^+^ T cells from three study groups as indicated was analyzed by the real-time RT-PCR for HCV and GAPDH RNA quantification. The data are expressed as HCV RNA levels relative (%) to control (without SN treatment, which is defined as 100). The results are mean ±SD of triplicate cultures, representative of three repeated experiments using pooled CD56^+^ T SN from three study groups. Statistical significance was performed using student *t*-test. (B) Immunofluoresence analysis of HCV NS3 protein expression in Huh 7 cells. HCV NS3 protein expression in Huh7 cells treated with or without pooled CD56^+^ T SN from three study groups as indicated was determined by immunofluoresence staining with antibody against HCV NS3 (green). The nuclei were stained with Hoechst 33258 (blue). One representative result of 3 experiments is shown (200×).

Since IFN-γ is the major component responsible for the CD56^+^ T cell-mediated antiviral action [35], we investigated whether heroin use or heroin use plus HCV infection affect IFN-γ expression in CD56^+^ T cells. As shown in Fig. 2B, freshly isolated CD56^+^ T cells from the heroin users, either with or without HCV infection, had significantly lower levels of IFN-γ mRNA than those from the healthy subjects. However, there was no difference between the heroin users with HCV infection and those without HCV infection (Fig. 2B). To further determine the functional capacity of CD56^+^ T cells in the heroin users with or without HCV infection and the control subjects, we assessed the ability of IL-12-stimulated CD56^+^ T cells to produce IFN-γ. IL-12 plays a key role in the induction of IFN-γ by the immune cells including CD56^+^ T cells [36], [37]. When stimulated by IL-12, CD56^+^ T cells from HCV-infected heroin users produced significantly lower levels of IFN-γ at both mRNA (Fig. 3A) and protein (Fig. 3B) levels than those from the healthy subjects. However, although IL-12-activated CD56^+^ T cells from the heroin users without HCV infection also produced lower levels of IFN-γ at both mRNA (Fig. 3A) and protein (Fig. 3B) levels than those from the healthy subjects, these differences were not statistically significant (P = 0.1849 and P = 0.175, respectively) (Fig. 3). This impaired ability to produce IFN-γ by CD56^+^ T cells from the heroin users with or without HCV infection was confirmed by the observation that CD56^+^ T cells from those subjects had diminished ability to inhibit HCV JFH-1 replication in human hepatocytes comparing with the cells from the healthy subjects (Fig. 4A and 4B).

### 3. HCV Viral Loads Is Negatively Associated with IL-12-Induced IFN-γ Production

**Figure 5:**
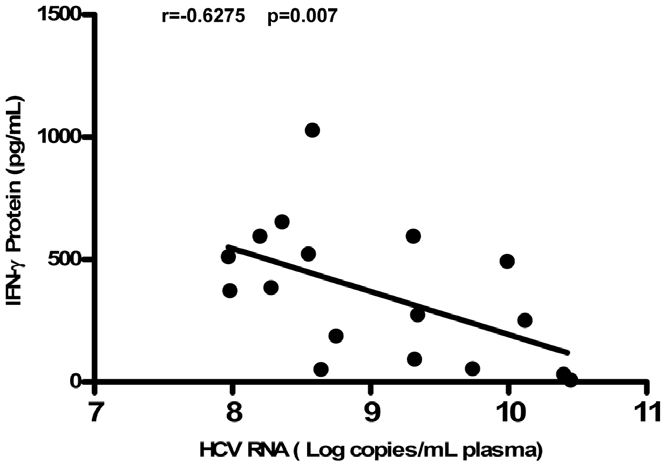
Plasma HCV viral loads is negatively associated with IFN-γ produced by IL-12-stimulated CD56^+^ T cells. Spearman correlation (r and p-value) was calculated to determine the relationship between the levels of IL-12-induced IFN-γ protein by CD56^+^ T cells and the levels of HCV RNA in plasma from HCV-infected heroin users (n = 17). The levels of IFN-γ protein produced by IL-12-stimulated CD56^+^ T cells were negatively correlated with those of plasma HCV RNA (P<0.01).

In order to further determine the negative impact of HCV infection on CD56^+^ T cells, we used linear regression analysis to determine whether there is a correlation between the plasma viral loads and the levels of IFN-γ proteins produced in IL-12-activated CD56^+^ T cells from the HCV-infected heroin users. As shown in Fig. 5, the plasma HCV viral loads is negatively associated with the levels of IFN-γ proteins produced by IL-12-stimulated CD56^+^ T cells from the HCV-infected heroin users (Fig. 5).

### 4. Effect of Heroin Use or Heroin Use plus HCV Infection on the Gene Expression of Key Elements and the Suppressors (SOCSs and PIASs) in IL-12 Pathway

**Figure 6:**
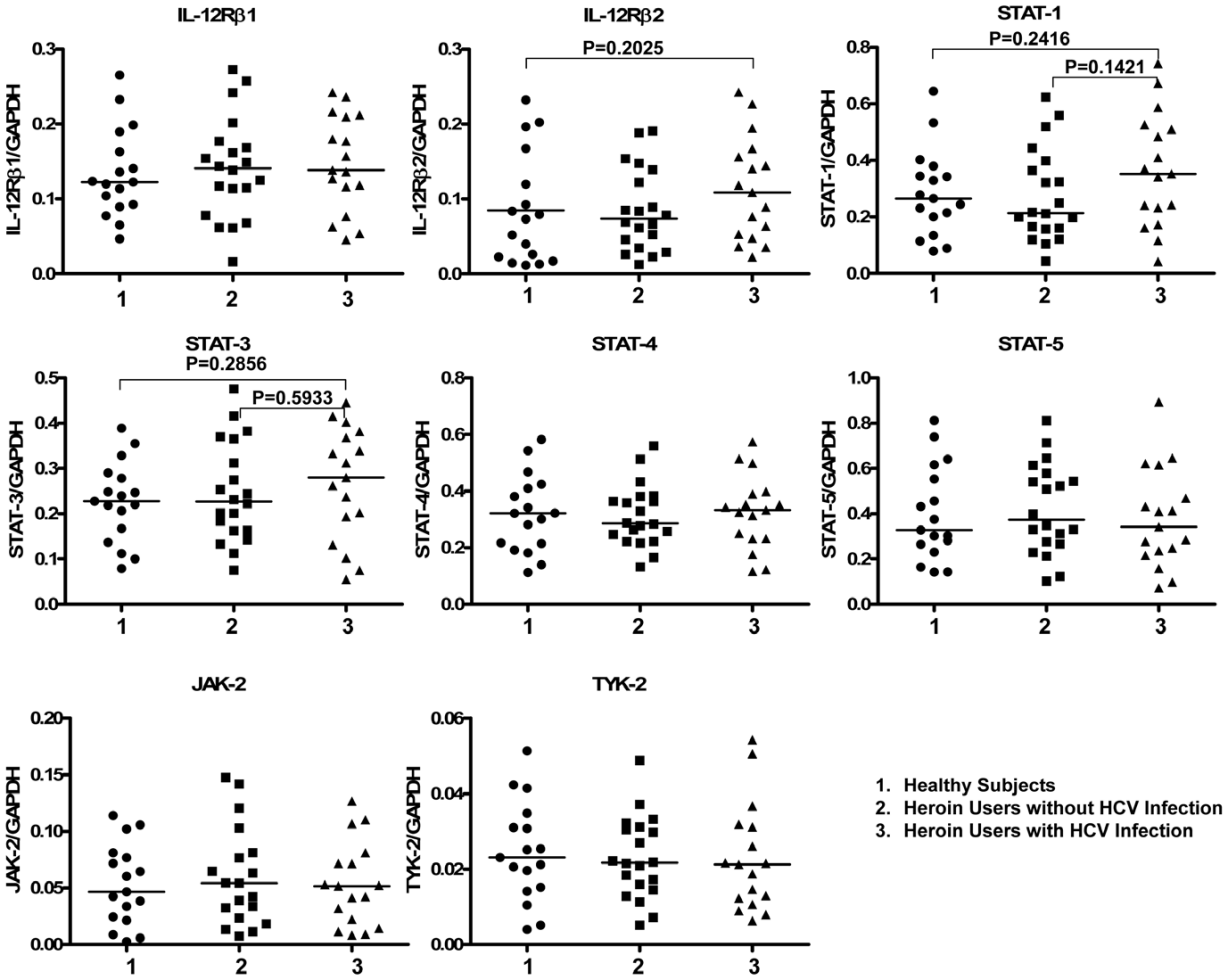
Effect of heroin use or heroin use plus HCV infection on the key element expression in IL-12 pathway. CD56^+^ T cells (unstimulated) were isolated from healthy subjects (n = 17) and heroin users with (n = 17) or without (n = 20) HCV infection. Total cellular RNA extracted from freshly isolated CD56^+^ T cells was subjected to the real-time RT PCR for key element (IL-12Rβ1, IL-12Rβ2, STAT-1, 3, 4, 5, JAK-2, TYK-2) in IL-12 pathway and GAPDH mRNA quantification. The data are expressed as the relative ratio of the key element mRNA level to GAPDH mRNA level. Median values are indicated by horizontal bars and statistical significance was calculated by the Mann-Whitney *U*-test.

**Figure 7:**
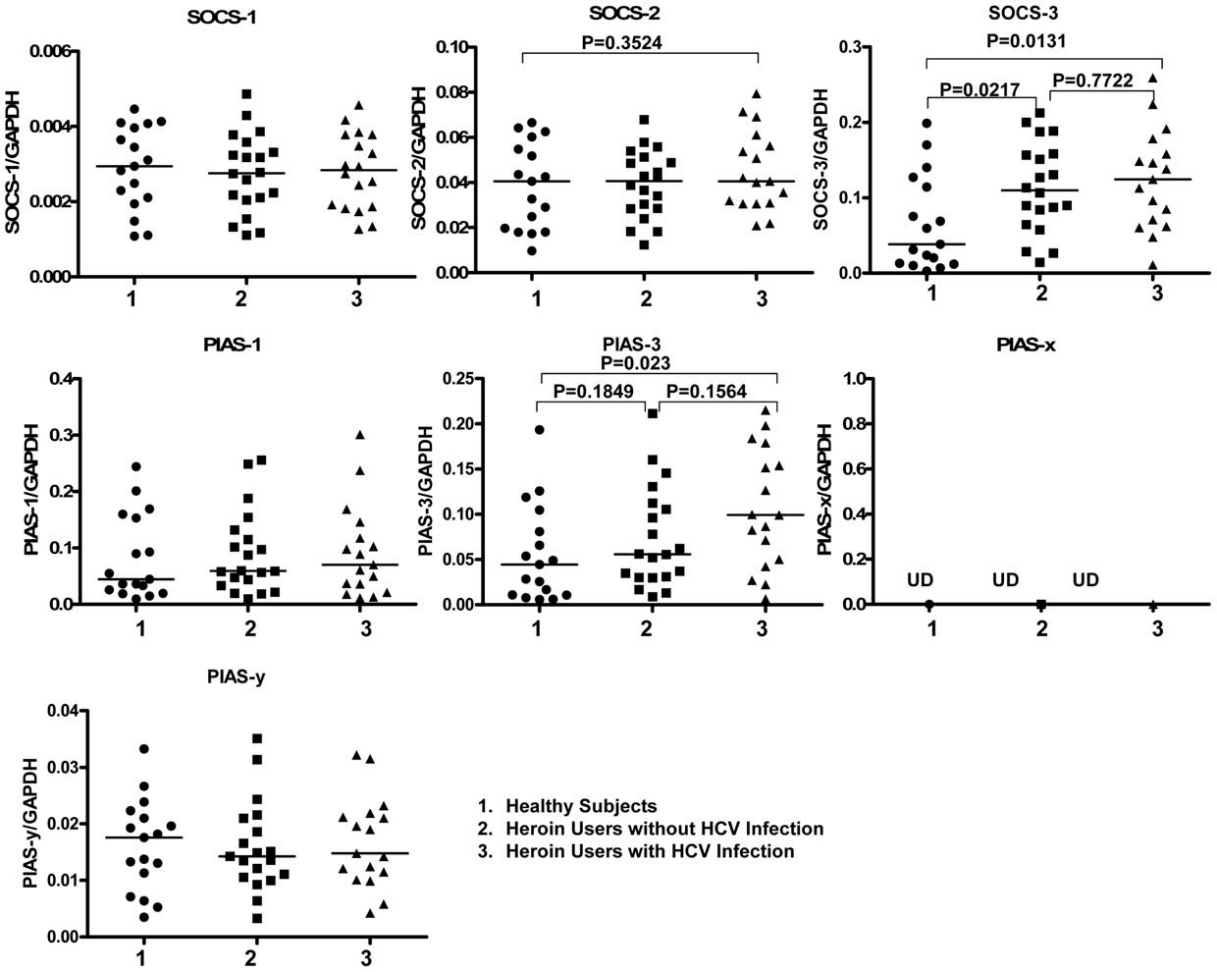
Heroin use or heroin use plus HCV infection induces the expression of the suppressors of IL-12 pathway. CD56^+^ T cells (unstimulated) were isolated from the healthy subjects (n = 17), heroin users with (n = 17) or without (n = 20) HCV infection. Total cellular RNA extracted from freshly isolated CD56^+^ T was subjected to the real-time RT PCR for the suppressor and GAPDH mRNA quantification. The data are expressed as the relative ratio of mRNA levels of suppressor (SOCS-1, 2, and 3, or PIAS-1, 3, x, and y) to those of GAPDH. Median values are indicated by horizontal bars and statistical significance was calculated by the Mann-Whitney *U*-test.

**Figure 8:**
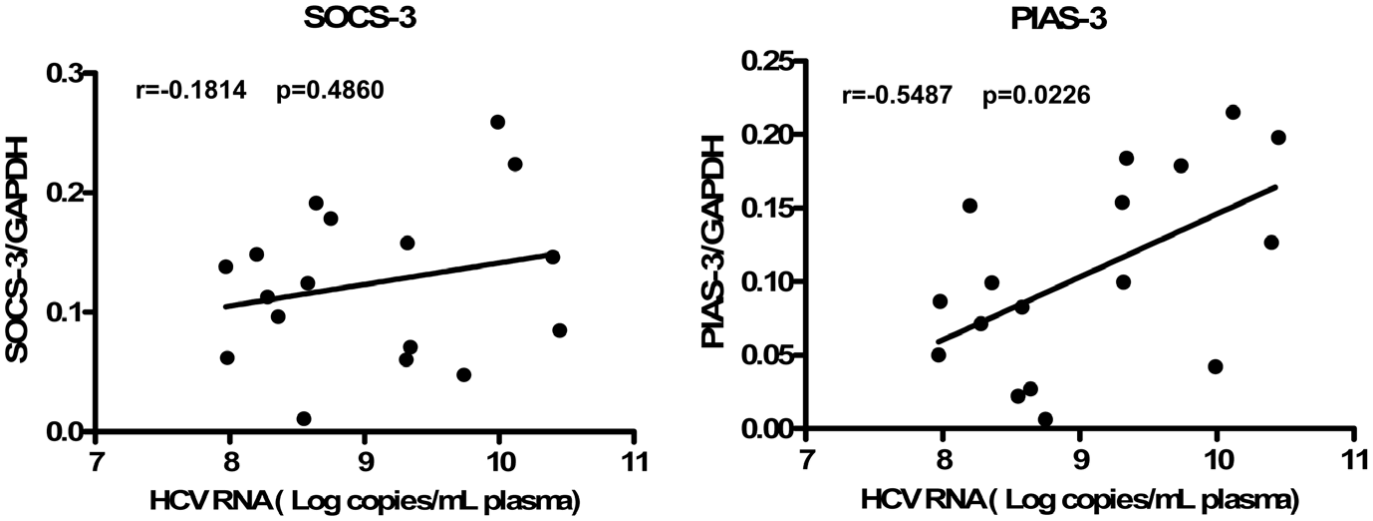
The relationship between plasma HCV viral loads and the levels of SOCS-3 and PIAS-3 in CD56^+^ T cells. Spearman correlation (r and p-value) was calculated to determine the relationship between the levels of SOCS-3/PIAS-3 in CD56^+^ T cells and the levels of HCV RNA in plasma from the heroin users infected with HCV (n = 17). The levels of PIAS-3 in CD56^+^ T cells were positively correlated with those of plasma HCV RNA (P<0.01).

To investigate whether heroin use or heroin use plus HCV infection has an impact on IL-12 pathway in CD56^+^ T cells, the transcriptional expression of key elements [IL-12 receptors, signal transducer and activator of transcription (STAT)-1, 3, 4, 5, janus kinase (JAK)-2, and tyrosine kinase (TYK)-2] in IL-12 pathway in CD56^+^ T cells was analyzed by the real-time RT PCR. The relative abundance of these elements in IL-12-stimulated CD56^+^ T cells were correlated to GAPDH mRNA and there was no difference in the expression patterns of these mRNAs between normal subjects, heroin users with HCV infection, and heroin users without HCV infection (P>0.05) (Fig. 6). To further determine the mechanism(s) involved in heroin use- or heroin use plus HCV infection-mediated dysfunction of CD56^+^ T cells, we examined whether heroin use or heroin use plus HCV infection was associated with the induction of intracellular suppressors of IL-12 signaling pathway. Among the suppressor members [suppressors of cytokine signaling (SOCS)-1, 2, and 3, protein inhibitors of activated STAT (PIAS)-1, 3, x, and y] tested, SOCS-3 levels, were elevated in CD56^+^ T cells freshly isolated from the heroin users with or without HCV infection (Fig. 7). In addition, CD56^+^ T cells freshly isolated from the HCV-infected heroin users had significantly higher levels of PIAS-3 than those from the healthy subjects (Fig. 7). Although there was an increase in PIAS-3 levels in CD56^+^ T cells from the heroin users without HCV infection, this difference was not statistically significant (Fig. 7). In addition, there was no significant difference in the expression of SOCS-3 and PIAS-3 between the heroin users without HCV infection and the heroin users with HCV infection (Fig. 7). Knowing that SOCS-3 and PIAS-3 were affected by heroin use or heroin use plus HCV infection, we further examined whether the expression of these two factors were associated with HCV viral loads. There was no association between the levels of SOCS-3 and HCV viral loads (Fig. 8). However, there is a positive association (P = 0.0226) between PIAS-3 and HCV viral loads (Fig. 8).

## Discussion

**Figure 9:**
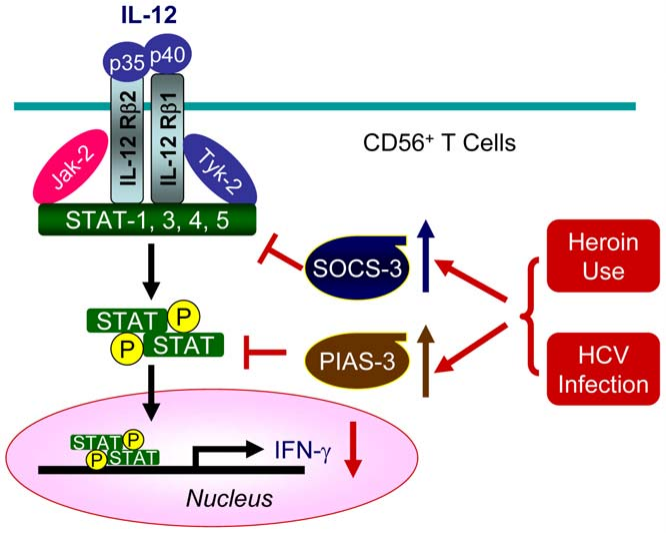
Schematic diagram of possible molecular mechanisms underlying heroin use/HCV infection-mediated suppression of IL-12 pathway. Although heroin use or heroin use plus HCV infection has little impact on the gene expression of key elements (IL-12Rβ1, IL-12Rβ2, STAT-1, 3, 4, 5, JAK-2, TYK-2) in IL-12 pathway in CD56^+^ T cells. Heroin use or heroin use plus HCV infection induces the expression of SOCS-3 or PIAS-3, the key negative regulators of IL-12 pathway, resulting in the downregulation of IL-12-mediated IFN-γ production.

In this study, we investigated the impact of heroin use or heroin use plus HCV infection on the antiviral functions of CD56^+^ T cells. As demonstrated in our earlier study [35], CD56^+^ T cells have the ability to inhibit HCV replication in human hepatocytes. This IFN-γ-mediated anti-HCV ability of CD56^+^ T cells, however, was impaired by heroin use or heroin use plus HCV infection (Fig. 4). We demonstrated that although there was no significant difference in CD56^+^ T cell frequency in PBMCs, CD56^+^ T cells from the heroin users with or without HCV infection had impaired ability to produce IFN-γ (Fig. 2). Despite of the limitation of lacking HCV-infected non-heroin group as a control, our study has shown that HCV infection was involved in the impairment of CD56^+^ T cell function, as we showed that when CD56^+^ T cells were stimulated with IL-12, significant decrease of CD56^+^ T cell produced IFN-γ was only observed in the HCV-infected heroin users (Fig. 3). The negative impact of HCV infection on CD56^+^ T cell function was also observed in the experiments showing that there is a negative association between HCV viral loads and the levels of IFN-γ produced by IL-12-stimulated CD56+ T cells (Fig. 5). To investigate the mechanism(s) underlying heroin use- or heroin use plus HCV infection-mediated downregulation of IFN-γ production in IL-12-stimulated CD56^+^ T cells, we examined whether heroin use or heroin use plus HCV infection have the impact on IL-12 pathway in CD56^+^ T cells. IL-12 is a central regulator that coordinates innate and adaptive immune responses [37], [38]. IL-12-mediated immune responses are largely through its ability to drive IFN-γ secretion. It is a potent inducer of IFN-γ from T, NK cells, as well as CD56^+^ T cells and is necessary for the development of Th1 response in most systems [36], [37]. Because IL-12 plays a central role in protection from diverse intracellular pathogens, IL-12 has, itself, been shown to be targeted by HCV [39]. IL-12 primarily signals through the JAK/STAT pathway to induce IFN-γ production (Fig. 9) [40]. IL-12p40 and p35 are IL-12 subunits that bind to IL-12 receptor, IL-12Rβ1 and β2, respectively. The binding brings the associated Jaks (Jak2 and Tyk2) phosphorylation and the activated signals are then transduced to STATs, which become phosphorylated and form dimers (Fig. 9). The STAT dimers shuttle into the nucleus where they bind to STAT-binding sites in the IFN-γ promoter and induce expression of IFN-γ gene (Fig. 9). We thus investigated whether heroin use or heroin use plus HCV infection suppress the expression of the key elements in JAK/STAT pathway. We first examined the gene expression of IL-12 Rβ1/β2 by CD56^+^ T cells isolated from the subjects of three groups. No significant differences in IL-12Rβ1/β2 expression were found among three groups (Fig. 6). We then focused on the members (STAT-1, 3, 4, and 5) of STAT family as well as JAK-2 and TYK-2, as the activation of these factors results in the production of IFN-γ.However, there were no significant differences in the expression of these positive regulators in CD56^+^ T cells isolated from the subjects of three groups (Fig. 6).

To further explore the mechanisms involved in heroin use- or heroin use plus HCV infection-mediated impairment of CD56^+^ T cell function, we attempted to determine whether heroin use or heroin use plus HCV infection induce the expression of negative regulators in IL-12 signaling pathway. Two suppressor families, SOCSs and PIASs, have been identified as the negative regulators in IL-12 signaling pathway [38], [40], [41], [42], [43] (Fig. 9). Among the suppressor family members examined, the level of SOCS-3 gene expression was elevated in CD56^+^ T cells isolated from the heroin users with or without HCV infection (Fig. 7). SOCS-3 has been reported to be a negative regulator of IL-12 signaling by preventing STAT-4 binding to the IL-12Rβ2 subunit [42]. SOCS-3 and SOCS-1, another member of SOCS family, have been shown to inhibit IFN-mediated antiviral activities [44] and IFN signaling [45], [46]. In addition to the mechanisms of SOCS family, we also examined the impact of heroin use or heroin use plus HCV infection on the expression of PIASs. The members of PIAS are a special inhibitors of STATs that inhibit STAT-mediated gene activation by blocking the DNA binding activity of STATs [47], [48]. PIAS members are also an important factors in the IL-12-induced IFN-γ production in peripheral blood T cells [49], [50]. We found that although heroin use or heroin use plus HCV infection had little effects on the expression of PIAS-1, PIAS-x and PIAS-y in CD56^+^ T cells, the levels of PIAS-3 gene expression in CD56^+^ T cells from the HCV-infected heroin users was significantly increased, comparing with the control subjects (Fig. 7). This observation was further confirmed by the correlation analyses, showing that there was a positive association between the levels of PIAS-3 and HCV viral loads (Fig. 8). These findings provide additional evidence to support the involvement of HCV infection in the impairment of CD56^+^ T cell function.

Taken together, the findings presented in this study provide compelling *ex vivo* as well as *in vivo* evidence that heroin use or heroin use plus HCV infection, through the induction of two key suppressors (SOCS-3 and PIAS-3) in IL-12 pathway, suppressed IFN-γ production by CD56^+^ T cells. This impaired ability of CD56^+^ T cells to produce IFN-γ was correlated with increased levels of HCV RNA in plasma from the HCV-infected heroin users. This *in vivo* finding is supported by our *in vitro* observation [35] that CD56^+^ T cells, through the secretion of IFN-γ that activates JAK/STAT pathway, inhibit HCV replication in human hepatocytes. Since CD56^+^ T cells are abundant in liver and a key component of host innate immune cell-mediated defense mechanisms, the impairment of CD56^+^ T cell function is likely to favor HCV infection and persistence in liver. It is known that heroin addicts are a high-risk group for HCV infection and the development of chronic HCV disease. Thus, to understand the impact of opioids such as heroin on the host immune system against HCV infection is of great interest for developing intervention strategies to reconstitute the immune system and to control viral replication in HCV-infected heroin users.

## Materials and Methods

### Ethics Statement

The Institutional Review Boards at The University of Pennsylvania School of Medicine approved this study, and written informed consent was obtained from all the study subjects. All investigations have been conducted according to the principles expressed in the Declaration of Helsinki.

### Study Population

Subjects addicted to heroin and admitted to the Clinic, The Center for Studies of Addiction at University of Pennsylvania School of Medicine, were recruited for this study. 37 heroin users and 17 healthy subjects were recruited for this study. Out of 37 heroin users, 17 were infected with HCV. Subjects were excluded if they had chronic systemic illness (cardiac, renal, pulmonary, hepatic, endocrine, metabolic or autoimmune disorders), major psychiatric disorders or if they were abusing other substrates other than heroin. For women, pregnancy was also a reason for exclusion. Control subjects were recruited using convenience sampling from the community in which the study site was located. Control subjects with no history of drug or alcohol abuse were also excluded if they had major medical or psychiatric disorders. The demographic data of these subjects are shown in Table 1.

**Table 1:** Demographics of Study Subjects.

Category		Normal Subjects (n = 17)	Heroin Users HCV^neg^ (n = 20)	Heroin Users HCV^pos^ (n = 17)
Age	Mean ± SD	39.4±10.2	38.8±12.0	44.1±11.3
	Range	24–55	22–60	27–62
Gender	Male/Female	10/7	13/7	11/6
Race	Caucasian/African American/others	8/6/3	10/8/2	7/9/1

### Reagents

Recombinant IL-12 was purchased from R&D Systems Inc. (Minneapolis, MN). Mouse anti-HCV NS3 antibody was kindly provided by Dr. Guangxiang Luo (University of Kentucky College of Medicine, Lexington, KY). The secondary antibodies used for Western blot (horseradish peroxidase-conjugated goat-anti-rabbit IgG, goat-anti-mouse IgG, and donkey anti-goat IgG) and Immunofluorescence (FITC-conjugated goat anti–mouse IgG antibody) were purchased from Jackson ImmunoResearch Laboratories, Inc. (West Grove, PA) and SouthernBiotech Inc. (Birmingham, AL), respectively.

### Isolation of CD56^+^ T Cells from PBMCs

PBMCs were isolated from peripheral blood by use of lymphocyte separation medium (Amersham Pharmacia Biotech) as described previously [51]. PBMCs were then subjected to CD56^+^ T cell isolation by CD3^+^CD56^+^ T Cell Isolation Kit (Miltenyi Biotec, Auburn, CA), in accordance with the manufacturer's instructions. The purity (% of CD3^+^CD56^+^) of isolated cells measured by flow cytometry was greater than 95% (Fig. 1). Purified CD56^+^ T cells were then cultured in RPMI 1640 containing 10% fetal bovine serum (HyClone, Logan, UT) and activated by IL-12 (20ng/mL) for 48h. Cell-free supernatants (SN) collected from the IL-12-activated CD56^+^ T cell cultures were used as CD56^+^ T SN.

### CD56^+^ T SN Treatment of HCV (JFH-1)-Infected Human Hepatocytes

The generation of infectious HCV Japanese fulminant hepatitis-1 (JFH-1) and infection of human hepatocytes (Huh 7 cells) were carried out as previously described [52]. For the experiments using CD56^+^ T SN treatment of hepatocytes infected with HCV JFH-1, SN was collected and pooled from IL-12-stimulated CD56^+^ T cell cultures of different subjects in each of the study groups. Pooled CD56^+^ T SN from each of the study groups was added to the hepatocyte cultures at day 3 postinfection and maintained in the cultures for 48h. CD56^+^ T SN-treated hepatocytes were then analyzed by immunofluorescent evaluation of HCV NS3 protein expression or by the real-time RT PCR for HCV RNA quantification. The details of these assays are described below.

### Quantitative Real-Time RT-PCR

Total cellular RNA extraction and the reverse transcription were performed as described [53]. The real-time RT-PCR for the quantification of HCV, IFN-γ, IL-12 receptor (IL-12Rβ1 and IL-12Rβ2), STAT-1, 3, 4, 5, JAK-2, TYK-2, SOCS-1, 2, 3, PIAS-1, 3, x, y and GAPDH mRNA was performed with the iQ SYBR Green Supermix (Bio-Rad Laboratories, Hercules, CA). The levels of GAPDH mRNA were used as an endogenous reference to normalize the quantities of target mRNA. The specific oligonucleotide primers used in this study are listed in Table 2. The primers were synthesized by Integrated DNA Technologies Inc. (Coralville, IA).

**Table 2:** Primers for Real-Time RT-PCR.

Primer	Accession No.	Orientation	Sequence (5′-3′)	Product Size (bp)
GAPDH	NM002046	Sense:	GGTGGTCTCCTCTGACTTCAACA	127
		Antisense:	GTTGCTGTAGCCAAATTCGTTGT	
HCV	NC004102	Sense:	RAYCACTCCCCTGTGAGGAAC	308
	NC009823	Antisense:	TGRTGCACGGTCTACGAGACCTC	
IFN-γ	NM000619	Sense:	AGAAAAATAATGCAGAGCCAAATT	275
		Antisense:	TGACTCCTTTTTCGCTTCCCTGTT	
IL-12Rβ1	NM153701	Sense:	CCAGGAACCAGACAGAGAAG	151
	NM005535	Antisense:	TCAGCACCAACCTGGTTATC	
IL-12Rβ2	NM001559	Sense:	ACATTCTTGGACATAGTGAGGCC	80
		Antisense:	GTACATCTGCTCACGGAAGCC	
Jak-2	NM004972	Sense:	TTTCGATGGATTTTGCCATT	251
		Antisense:	GCGAACAGTTTCCATCTGGT	
Tyk-2	NM003331	Sense:	CTTGACTGTGAACCGGGACT	248
		Antisense:	TGATGTGCTCGTGGTAGAGC	
STAT-1	NM139266	Sense:	GTGGAAAGACAGCCCTGCAT	67
	NM007315	Antisense:	ACTGGACCCCTGTCTTCAAGAC	
STAT-3	NM003150	Sense:	GGCCCCTCGTCATCAAGA	59
		Antisense:	TTTGACCAGCAACCTGACTTTAGT	
STAT-4	NM003151	Sense:	CACCTGCCACATTGAGTCAACTA	72
		Antisense:	TAAGACCACGACCAACGTACGA	
STAT-5a, 5b	NM003152	Sense:	AGATGCTGGCCGAGGTCAAC	212
	NM012448	Antisense:	AGACTTGGCCTGCTGCTCAC	
SOCS-1	NM003745	Sense:	GACGCCTGCGGATTCTACTG	138
		Antisense:	GGCCATCTTCACGCTAAGGG	
SOCS-2	NM003877	Sense:	TGCAAGGATAAGCGGACAGG	104
		Antisense:	CAGAGATGCTGCAGAGATGG	
SOCS-3	NM003955	Sense:	TGCGCCTCAAGACCTTCAGC	227
		Antisense:	GATGCGCAGGTTCTTGGTCC	
PIAS-1	NM016166	Sense:	TCCCACCCAATCTTTGTGTG	380
		Antisense:	GCCGCATTTTACCAAGTGGA	
PIAS-3	NM006099	Sense:	TGCTGGCCGGAACAAGAGTG	172
		Antisense:	AGGGGGCAAAGAGAGAAGGG	
PIAS-x (a,b)	NM173206	Sense:	TCTTCTGACGAAGAGGAAGACC	275
	NM004671	Antisense:	TCAGAAGATGTTCCAAGCTTCA	
PIAS-y	NM015897	Sense:	GAGAAGAAGCCCACCTGG A	99
		Antisense:	ACACTCGCTCAGGATCTTCG	

### HCV Viral Load

Total RNA from plasma was extracted with Tri-Reagent-BD (Molecular Research Center) in accordance with the manufacturer's instructions. The real time RT-PCR assay that we have developed [15], [54] with minor modifications was used for the quantification of HCV RNA. HCV genome primers used in this study are listed in Table 2 and the real time RT-PCR was performed with the iQ SYBR Green Supermix. A standard curve of HCV was generated with 10-fold dilutions of HCV 5′non-coding region RNA control that had been pre-quantified by a spectrophotometer (Eppendorf Scientific, Inc. Westbury, NY). Thermal cycling conditions were designed as follows: initial denaturation at 95°C for 3 minutes, followed by 40 cycles of 95°C for 10 seconds and 60°C for 1 minute.

### Immunoassays

The enzyme-linked immunosorbent assay (ELISA) for IFN-γ was performed according to the protocol provided by the manufacturer (PBL Biomedical Laboratories, Piscataway, NJ). Immunofluorescent evaluation of HCV NS3 protein in HCV-infected hepatocytes was carried out as previously described [53]. Briefly, HCV-infected hepatocytes were fixed with 4% paraformaldehyde for 20 min at room temperature on glass coverslips and then pretreated with a blocking solution for 30 min. The coverslips were then incubated with anti-NS3 antibody (1∶1000) in blocking solution at room temperature for 60 min and were subsequently incubated with FITC-conjugated goat anti–mouse IgG antibody (1∶200) for 30 min and Hoechst 33258 (for nuclei) for 15 min. The coverslips were washed 5 times with 1×PBS, mounted in Vectorshield (Vector Laboratories, Burlingame, CA), and viewed with a fluorescence microscope (Olympus 1X71 Inverted Microscope, Japan).

### Statistical Analysis

Results were expressed as medians or mean ± standard deviations and statistically analyzed using Mann-Whitney *U*-test or paired Student's t test (Prism 4.0 software) where appropriate. Correlations between the IL-12-induced IFN-γ by CD56^+^ T cells and the levels of HCV RNA in plasma, or between the levels of SOCS-3/PIAS-3 in CD56+ T cells and the levels of HCV RNA in plasma was performed using Spearman's rank test (Prism 4.0 software). Statistical significance was defined as P<0.05.
